# Supplementary figures and images for: Structure and function of yeast Lso2 and human CCDC124 bound to hibernating ribosomes
Source: PLoS Biol. 2020 Jul 20;18(7):e3000780. doi: 10.1371/journal.pbio.3000780 (PMC7392345; doi:10.1371/journal.pbio.3000780)

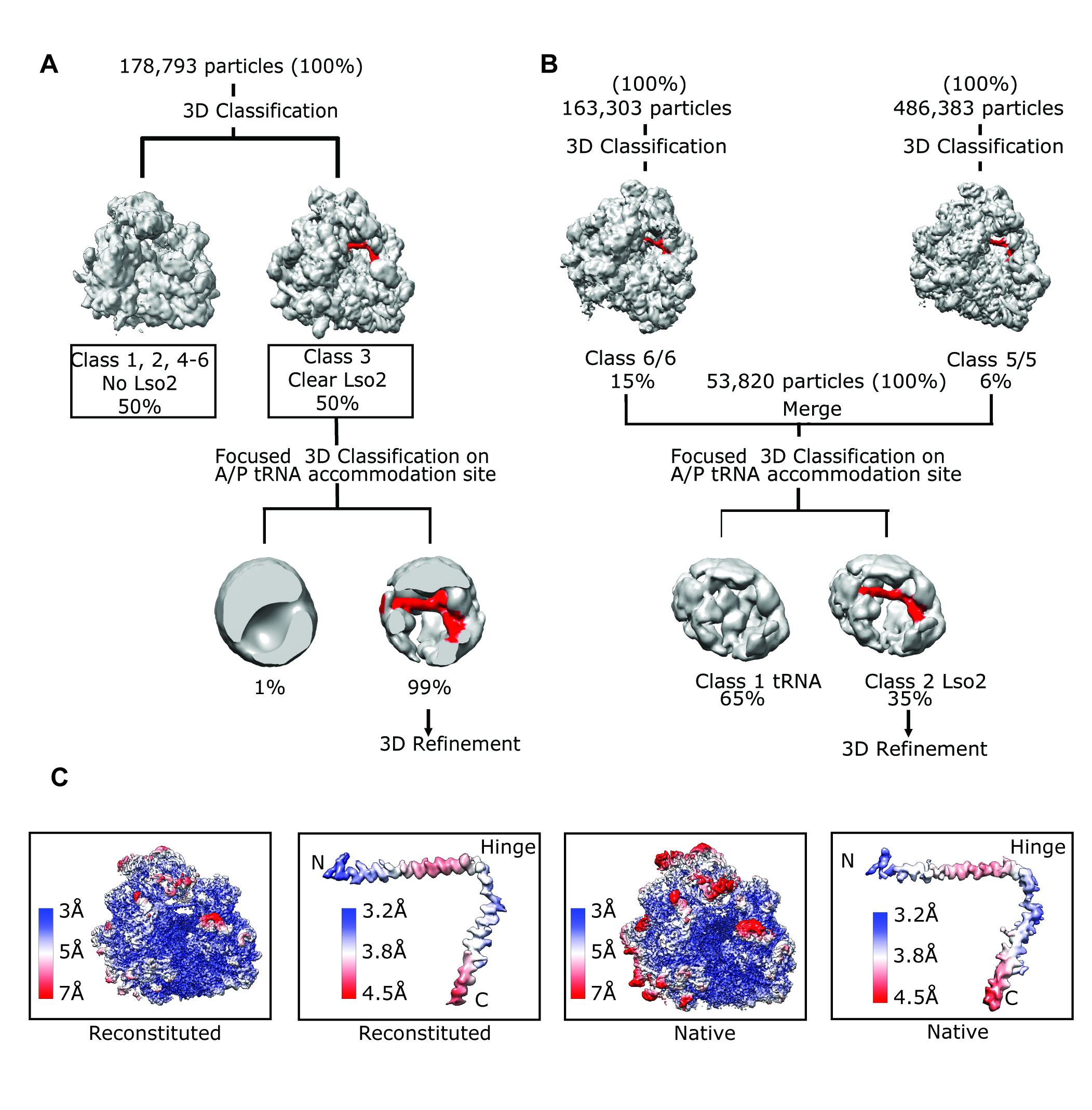

Supplement: S1 Fig — (A–B) Sorting schemes for reconstituted (A) and native (B) Lso2–80S reconstructions. For the native Lso2–80S structure, particles containing Lso2 were merged from 2 individual collections after 3D classification because of relatively low occupancy with Lso2 (5% and 16% of all particles, respectively). For both reconstituted and native complexes, the Lso2-containing classes were locally classified using an ellipsoid mask covering the A- and P-site tRNA binding sites of the 80S. The Lso2-containing classes were refined to an overall resolution of 3.5 Å and 3.4 Å, respectively. (C) The local resolution range for 80S ribosome and isolated Lso2 is indicated. A, acceptor; Lso2, late-annotated short open reading frame 2; P, peptidyl. (TIF) [file pbio.3000780.s001.tif]

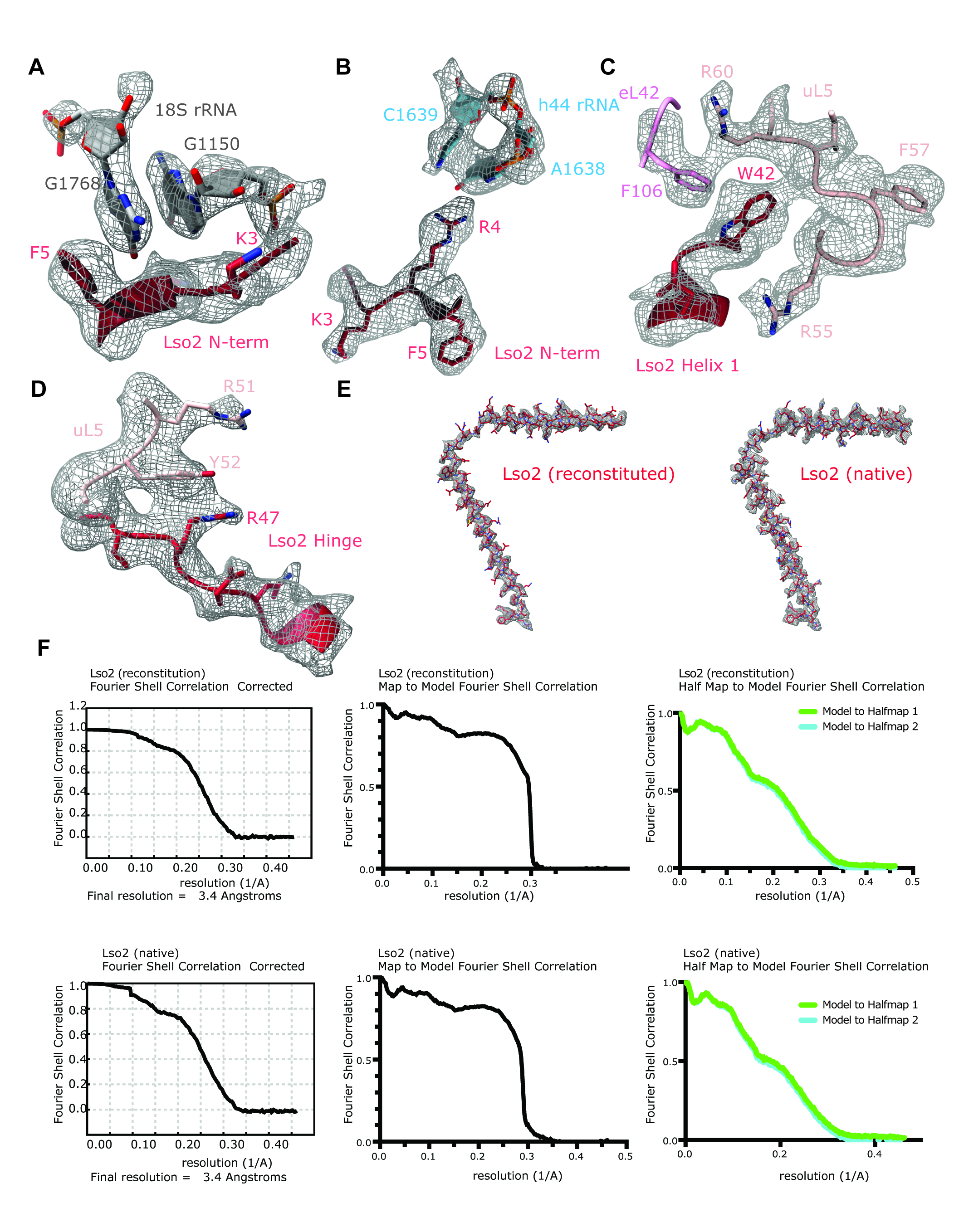

Supplement: S2 Fig — (A) View highlighting the model for the N-terminus of Lso2 interacting with rRNA helix h28 (G1150) and h45 (G1768) fitting into respective densities (transparent gray mesh). (B) View focusing on the Lso2 N-terminal R4 interacting with h44. (C) View focusing on the stacking of Lso2 hinge residue W42 inside a cleft formed by large subunit proteins eL42 and uL5. (D) View focusing on interactions between the hinge region of Lso2 (R47) and uL5 residues. (E) Fit of the entire Lso2 model into isolated density. (F) Overall FSC curves, map to model FSC curves, and half map to model FSC curves for validation of the Lso2–80S model fitting the reconstituted and native Lso2–80S reconstructions. FSC, Fourier shell correlation; Lso2, late-annotated short open reading frame 2 (TIF) [file pbio.3000780.s002.tif]

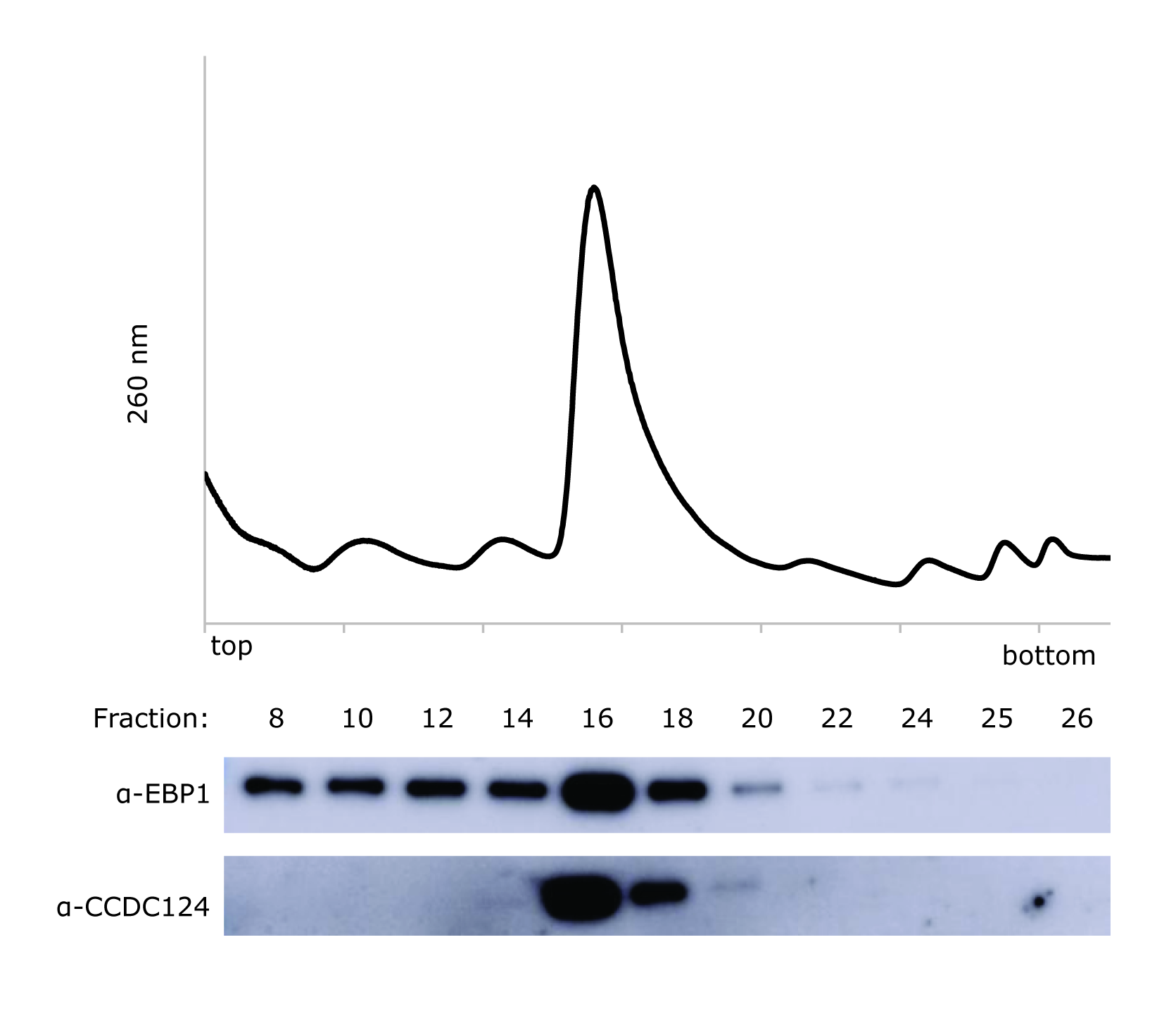

Supplement: S3 Fig — HEK293T cell lysates were fractionated through 10%–40% sucrose gradients. Fractions were collected, and western blotting using antibodies against CCDC124 and EBP1 was performed. Both factors were found to associate with 80S ribosomes. CCDC124, coiled-coil domain containing short open reading frame 124; EBP1, ErbB3-binding protein 1; HEK, human embryonic kidney. (TIF) [file pbio.3000780.s003.tif]

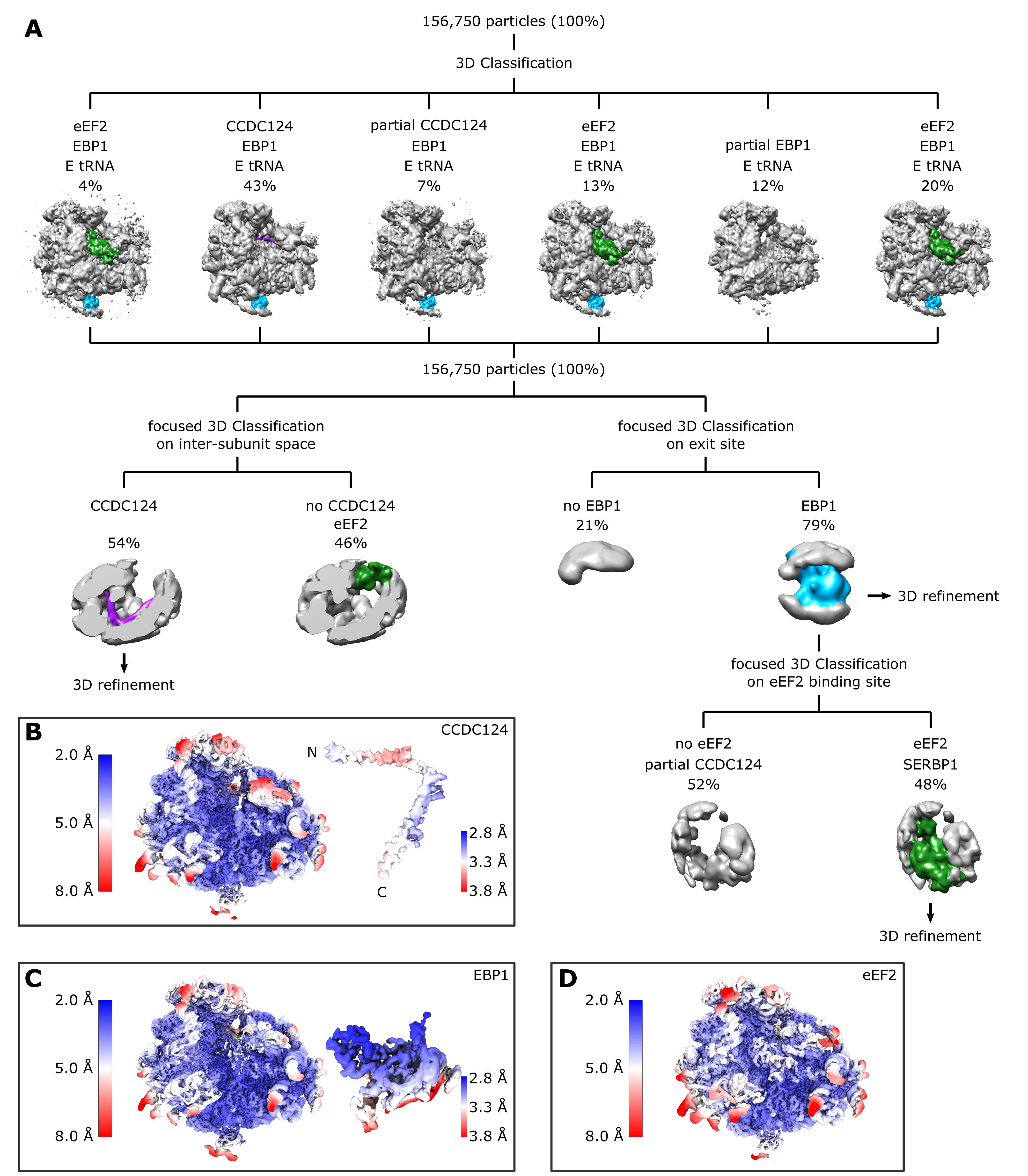

Supplement: S4 Fig — (A) In a first round of 3D classification, particles were separated, containing E-site tRNA and EBP1 and either CCDC124 (in the nonrotated state) or SERBP1 and eEF2 (in the rotated-2) state. Independent subclassifications using a regional mask were then performed using ellipsoid masks covering the A- and P-site tRNA binding sites (for CCDC124 and eEF2/SERBP1, respectively) or on the peptide exit site (for EBP1). This yielded homogenous ribosome classes containing exclusively either CCDC124 and EBP1 or SERBP1/eEF2 and EBP1. The classes displaying CCDC124–80S, SERBP1/eEF2–80S and EBP1-enriched SERBP1/eEF2–80S were refined to overall resolution of 3.0 Å, 3.1 Å, and 2.9 Å. (B–D) Local resolution ranges for CCDC124–80S (B), EBP1–80S (C), and eEF2/SERPB1–80S (D) and isolated CCDC124 and EBP1 are indicated. A, acceptor; CCDC124, coiled-coil domain containing short open reading frame 124; EBP1, ErbB3-binding protein 1; eEF2, eukaryotic elongation factor 2; P, peptidyl; SERBP1, SERPINE1 mRNA-binding protein 1 (TIFF) [file pbio.3000780.s004.tiff]

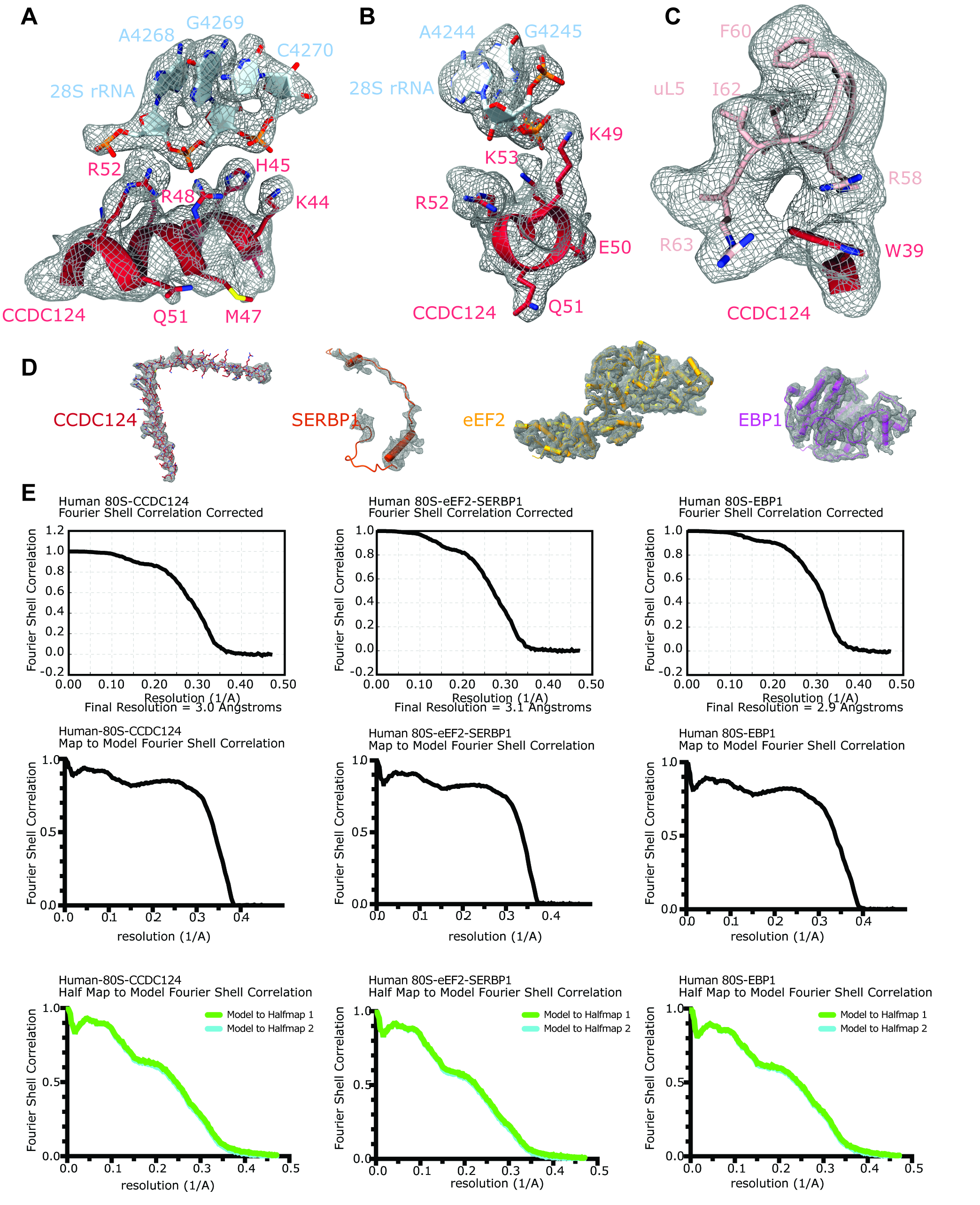

Supplement: S5 Fig — (A–B) View highlighting the hinge region of CCDC124 interacting with H85 (A) and H84 (B) of the 28S rRNA. (C) View focusing on the interaction of CCDC124 W39 stacking with residues of uL5 in a manner distinct from Lso2, wherein eL42 also participates in stabilizing Lso2 W42. (D) Fits of the entire CCDC124 and EBP1 models into the respective isolated densities. (E) Overall FSC curves, map to model FSC curves, and half map to model FSC curves for validation of the final CCDC124–80S, eEF2/SERPB1–80S, and EBP1–80S models fitting the respective densities. CCDC124, coiled-coil domain containing short open reading frame 124; EBP1, ErbB3-binding protein 1; FSC, Fourier shell correlation; Lso2, late-annotated short open reading frame 2; SERPB1, SERPINE1 mRNA-binding protein 1 (TIF) [file pbio.3000780.s005.tif]

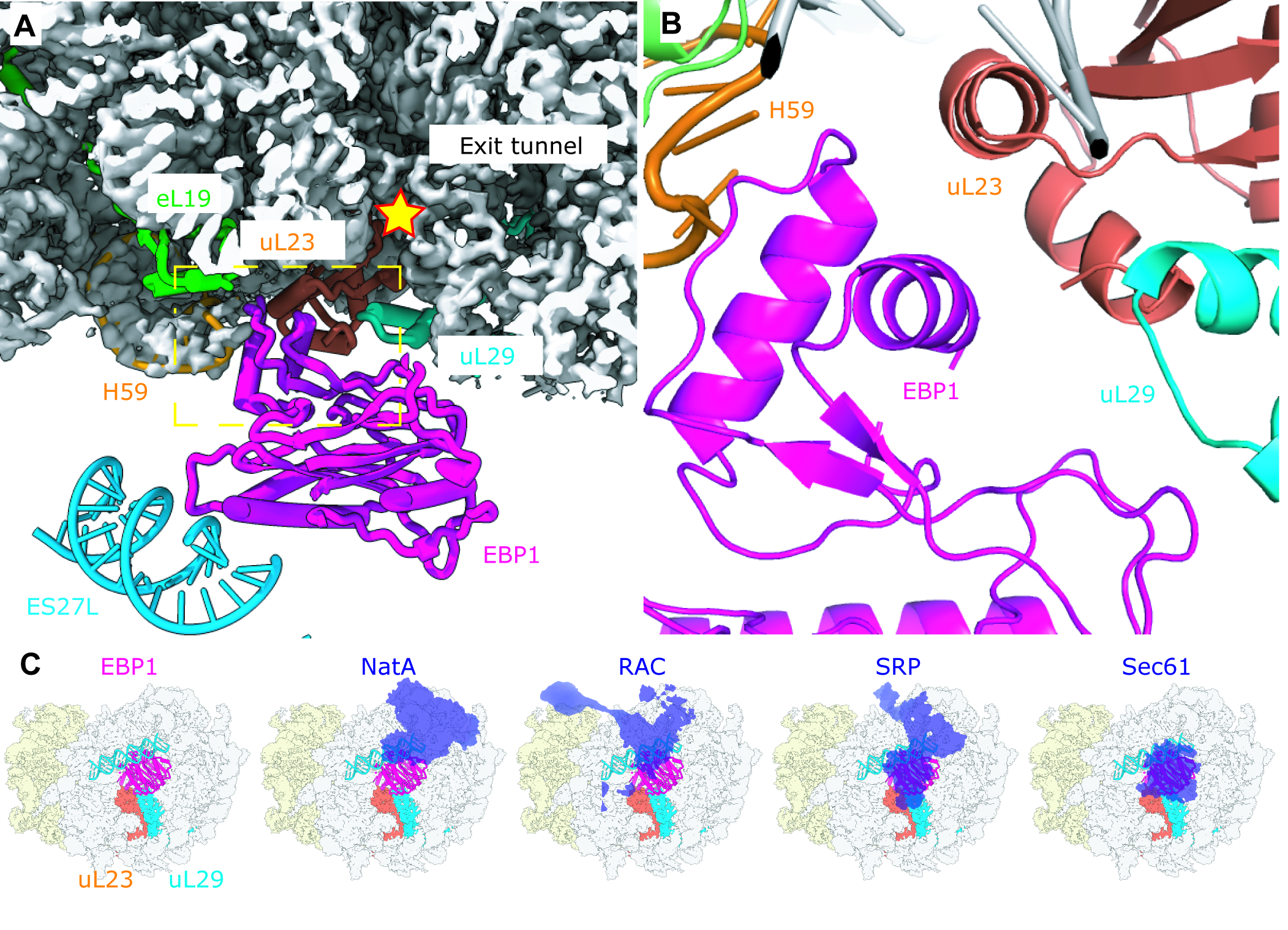

Supplement: S6 Fig — (A) Close-up view showing the overall positioning of EBP1 at the peptide exit site. (B) Zoom on the interaction between EBP1 and H59. (C) Comparison of the EBP1 position with other exit-site ligands. EBP1 binding would overlap with a majority of nascent chain-interacting factors such as NatA [43], RAC [40], SRP [69], or Sec61 [41,42,70]. EBP1, ErbB3-binding protein 1; NatA, N-α-Acetyltransferase; RAC, ribosome associated complex; Sec61, secretory protein 61; SRP, signal recognition particle. (TIF) [file pbio.3000780.s006.tif]

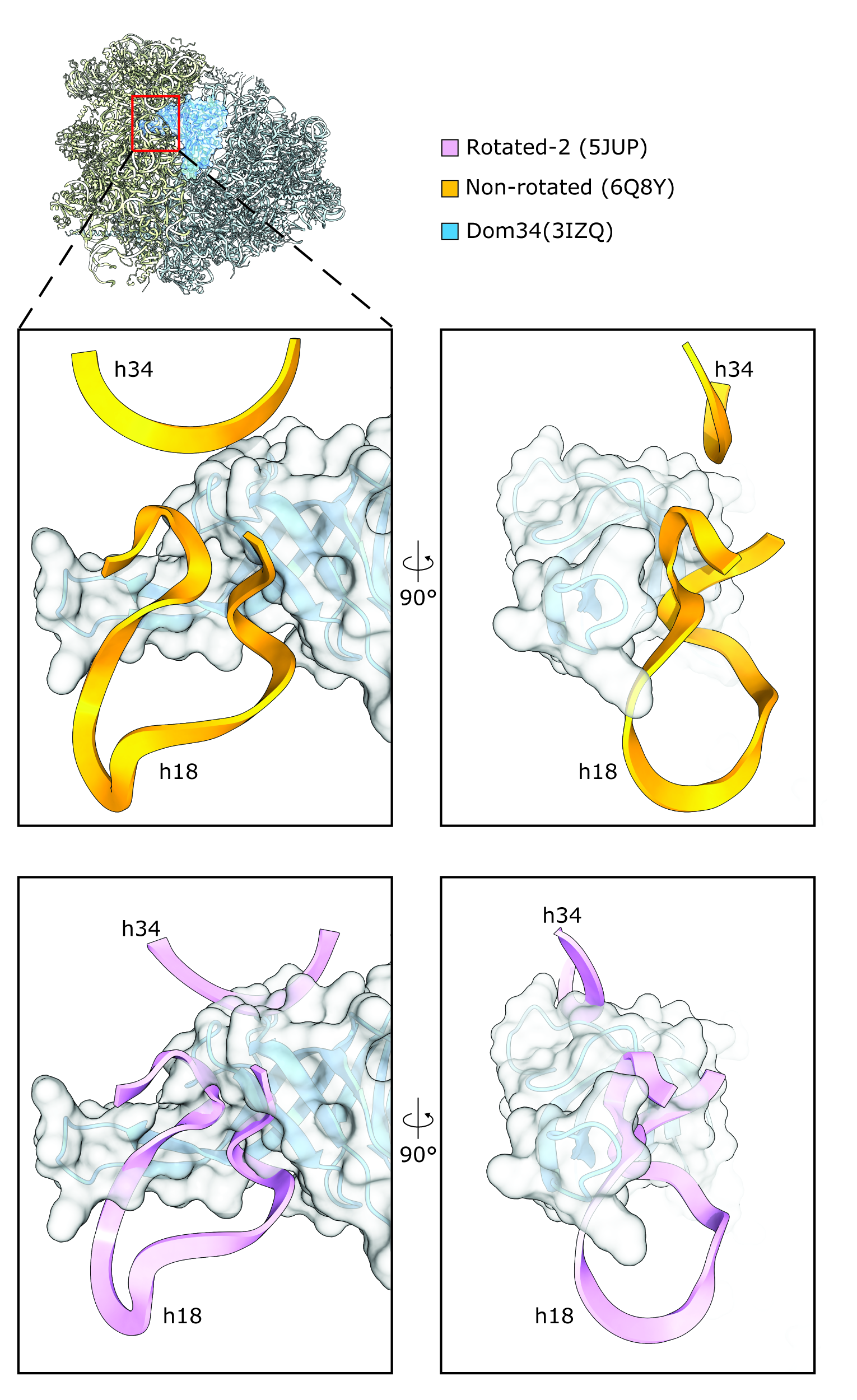

Supplement: S7 Fig — Dom34 in complex with Hbs1 (and also ABCE1) is usually found in the nonrotated state. The structure of a yeast 80S in the nonrotated state (PDB: 6Q8Y; representing Lso2–80S) [25] was superimposed with one in the rotated-2 state (PDB: 5JUP; representing Stm1–80S) [26] based on the 60S subunits. We note that the Dom34 N-terminal domain (taken from PDB: 3IZQ) [45] would clash with 18S rRNA helix h18 and h34 in the rotated-2 state. This indicates that the rotated (Stm1-containing) 80S ribosome cannot be split by the Dom34 splitting system. Dom34, duplication of multilocus region 34; Hbs1, Hsp70 subfamily B suppressor; Lso2, late-annotated short open reading frame 2; PDB, Protein Data Bank; Stm1, suppressor of target of Myb protein 1 (TIF) [file pbio.3000780.s007.tif]

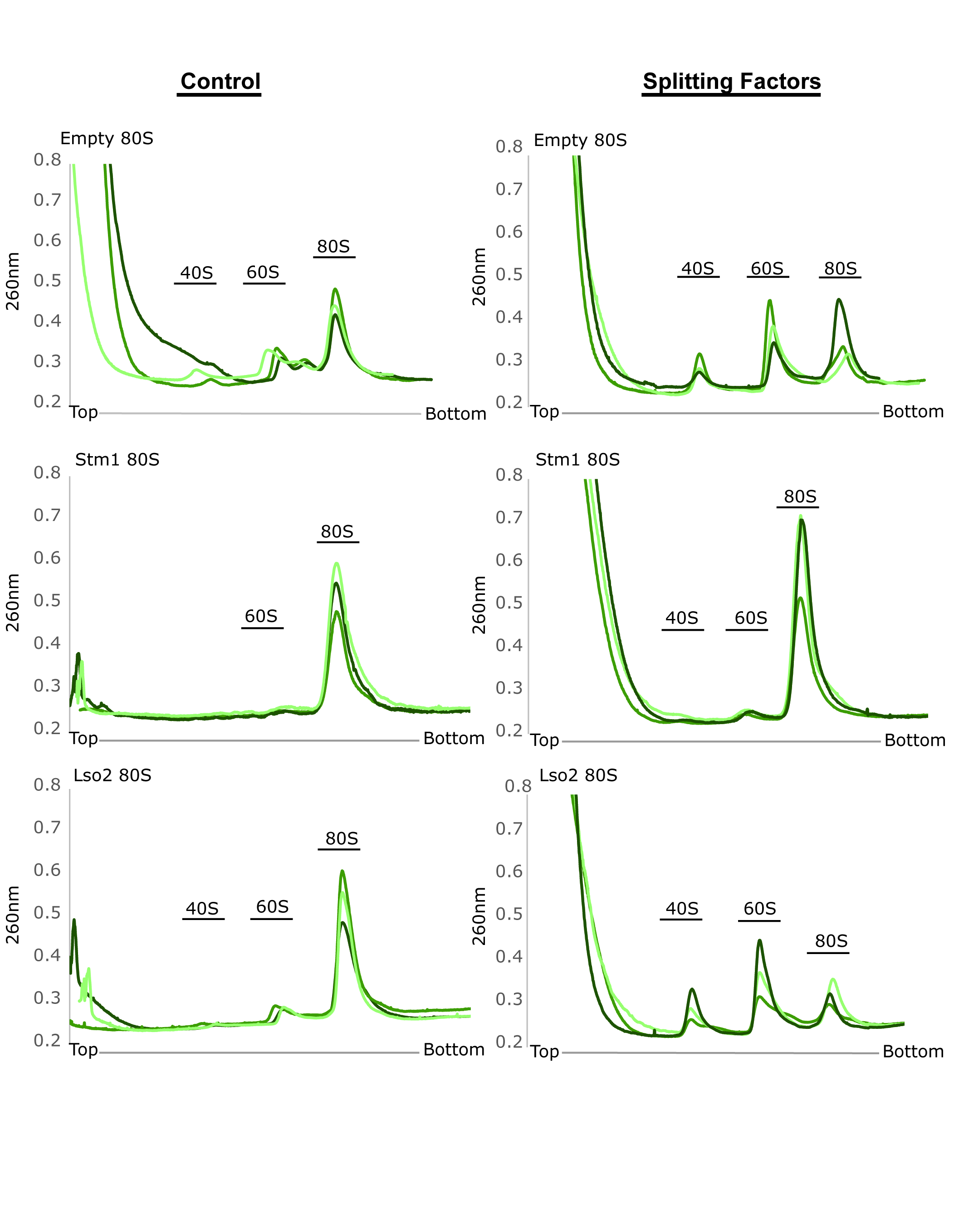

Supplement: S8 Fig — UV profiles for splitting assays. Assays contain 5 pmol of ribosomes (treated with puromycin or bound to Lso2 or bound to Stm1) and 25 pmol of factors (60S subunit anti-association factor eIF6, Dom34, Hbs1, and ABCE1). 50-μl reactions were spun through a 10%–50% sucrose gradient, and an absorption profile at 260 nm (A260) was recorded. All experiments were carried out in triplicates (as indicated in different shades of green). Raw data can be found in S1 Data. Dom34, duplication of multilocus region 34; eIF6, eukaryotic initiation factor 6; Hbs1, Hsp70 subfamily B suppressor; Lso2, late-annotated short open reading frame 2; Stm1, suppressor of target of Myb protein 1 (TIF) [file pbio.3000780.s008.tif]

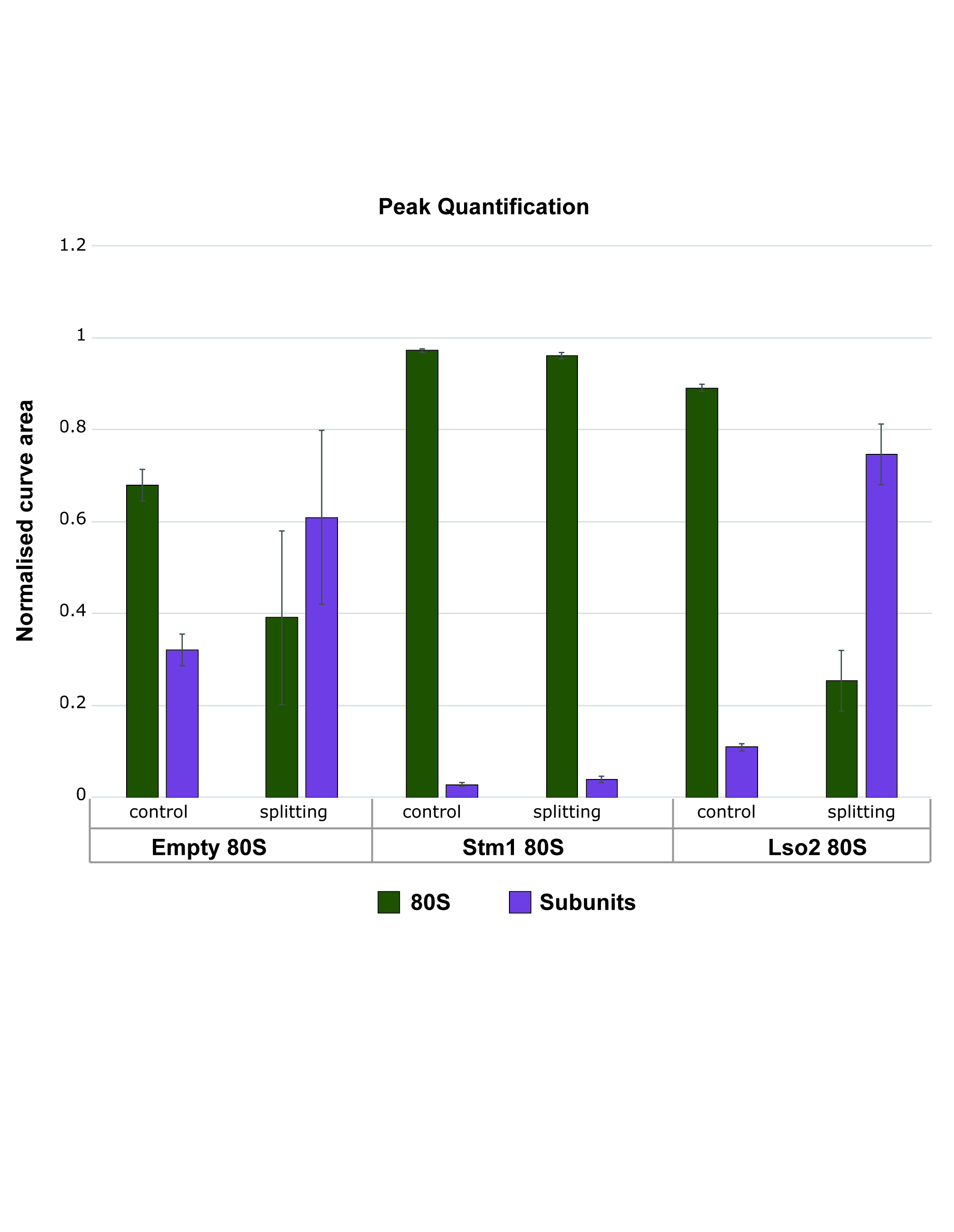

Supplement: S9 Fig — The relative abundance of 80S ribosomes and subunits in in vitro splitting assays was estimated by comparing the areas under A260 absorption peaks from 40S, 60S, and 80S subunits (calculated as described in Methods). Note that Stm1–80S have the highest relative abundance after splitting, whereas Lso2–80S show the lowest relative abundance after splitting. Calculations can be found in S2 Data. Lso2, late-annotated short open reading frame 2; Stm1, suppressor of target of Myb protein 1 (TIF) [file pbio.3000780.s009.tif]

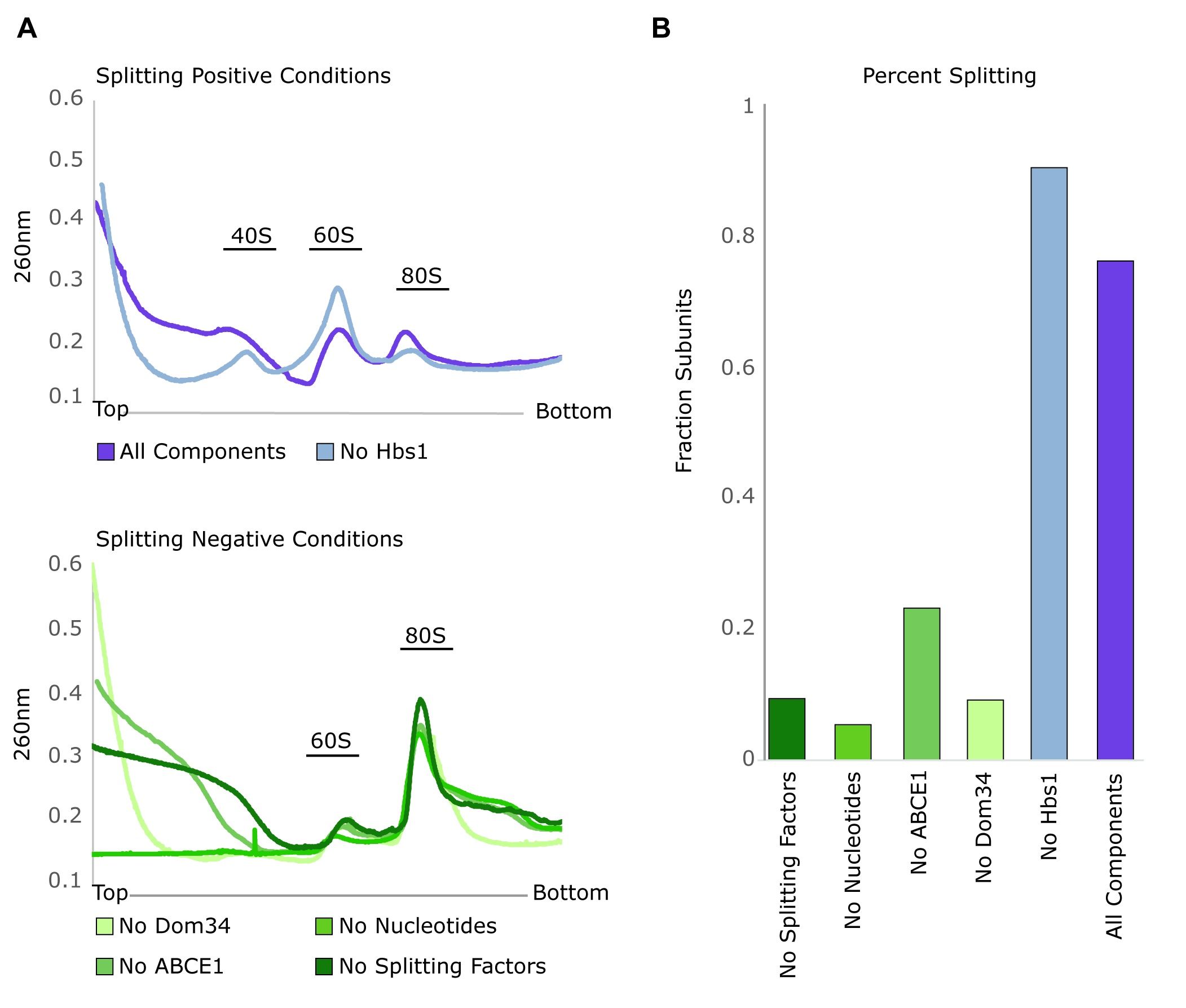

Supplement: S10 Fig — (A) In vitro splitting assays were assembled (materials and methods) with 80S ribosomes reconstituted with Lso2, omitting individual factors or nucleotides as annotated. UV profiles for splitting assays were collected following separation over a 10%–50% sucrose gradient; absorption profiles were collected at 260 nm. Splitting was observed in reactions when all components (eIF6, ABCE1, Hbs1, Dom34, 1 mM ATP, 1 mM GTP) were present or in the absence of Hbs1. Conversely, splitting was not observed when Dom34, Hbs1, or nucleotides were omitted from the reaction or in reactions containing eIF6 and nucleotides without other factors. (B) Percent splitting was calculated by comparing the areas under A260 absorption peaks from 40S, 60S, and 80S subunits (calculated as described in Methods) and comparing the relative abundance of 80S ribosomes and subunits. Raw data can be found in S3 Data. Dom34, duplication of multilocus region 34; eIF6, eukaryotic initiation factor 6; Hbs1, Hsp70 subfamily B suppressor; Lso2, late-annotated short open reading frame 2 (TIF) [file pbio.3000780.s010.tif]

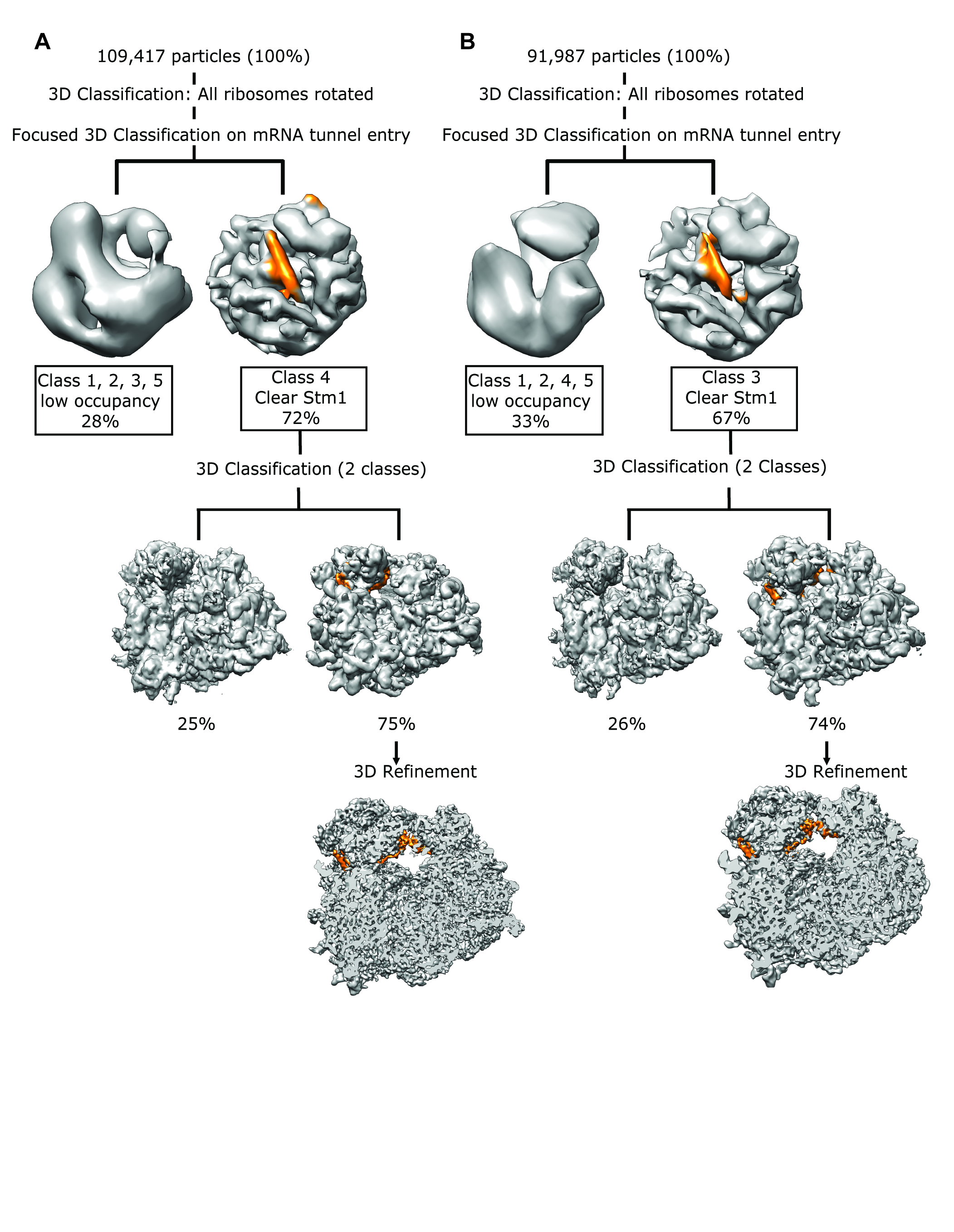

Supplement: S11 Fig — Stm1–80S were prepared following protocols previously reported [17] to enrich Stm1 binding and used in in vitro splitting reactions. Stm1–80S were collected from the sucrose gradient following in vitro splitting reactions with and without splitting factors (control). These 80S fractions were analyzed by cryo-EM in which particles were assessed for ribosome rotational states and presence of Stm1. (A) 80S ribosome population from the control splitting assay. (B) 80S ribosome population remaining following incubation with splitting factors. An initial 3D classification revealed that a vast majority of 80S are in the rotated state. These particles were further subjected to local classification using and ellipsoid mask covering the region of the mRNA channel of the 40S to classify for Stm1-containing particles (approximately 50%), which were further refined. Stm1 density is displayed in orange. cryo-EM, cryo-electron microscopy; Stm1, suppressor of target of Myb protein 1 (TIF) [file pbio.3000780.s011.tif]
