# Supplementary material for: Structure and function of yeast Lso2 and human CCDC124 bound to hibernating ribosomes
Source: PLoS Biol. 2020 Jul 20;18(7):e3000780. doi: 10.1371/journal.pbio.3000780 (PMC7392345; doi:10.1371/journal.pbio.3000780)
Supplement: S1 Table — Overview of cryo-EM data collection, data processing, and model-fitting parameters for yeast native and reconstituted Lso2–80S complexes and for human CCDC124–EBP1 and SERBP1–eEF2–EBP1 complexes. CCDC124, coiled-coil domain containing short open reading frame 124; cryo-EM, cryo-electron microscopy; EBP1, ErbB3-binding protein 1; eEF2, eukaryotic elongation factor 2; Lso2, late-annotated short open reading frame 2; SERBP1, SERPINE1 mRNA-binding protein 1 (DOCX) [file pbio.3000780.s012.docx]

|  | Yeast ribosome  +Lso2  (Native) | Yeast ribosome  +Lso2 (Reconstituted) | Human ribosome  +CCDC124/EBP1 | Human ribosome  +eEF2/SERBP1/EBP1 | Human ribosome  +EBP1 combined |
| --- | --- | --- | --- | --- | --- |
| **Data collection and processing** |  |  |  |  |  |
| Magnification | 75,000 | 75,000 | 75,000 | 75,000 | 75,000 |
| Voltage (kV) | 300 | 300 | 300 | 300 | 300 |
| Electron exposure (e–/Å^2^) | 28 | 28 | 28 | 28 | 28 |
| Defocus range (μm) | -1.1 to -2.3 | -1.1 to -2.3 | -0.9 to -3.0 | -0.9 to -3.0 | -0.9 to -3.0 |
| Pixel size (Å) | 1.084 | 1.084 | 1.061 | 1.061 | 1.061 |
| Symmetry imposed | *C1* | *C1* | *C1* | *C1* | *C1* |
| Initial particle images (no.) | 649,686 | 178,793 | 332,890 | 332,890 | 332,890 |
| Final particle images (no.) | 34,951 | 88,523 | 84,429 | 72,367 | 127,706 |
| Map resolution (Å)  FSC threshold | 3.5  0.143 | 3.4  0.143 | 3.0  0.143 | 3.1  0.143 | 2.9  0.143 |
|  |  |  |  |  |  |
| **Refinement** |  |  |  |  |  |
| Model resolution (Å)  FSC threshold | 3.5  0.5 | 3.4  0.5 | 3.0  0.5 | 3.0  0.5 | 2.9  0.5 |
| Map sharpening *B* factor (Å^2^) |  |  | 90 | 80 | 90 |
| Model composition  Non-hydrogen atoms  Protein residues  RNA | 196,487  11,063  5,105 | 196,487  11,063  5,105 | 222,325  12,128  5,864 | 228,957  12,949  5,876 | 228,587  12,938  5,864 |
| *B* factors (Å^2^)  Protein  RNA | 41.80  36.11  46.37 | 62.90  56.24  68.24 | 84.50  78.95  88.93 | 110.28  109.19  111.30 | 74.33  61.94  84.74 |
| R.m.s. deviations  Bond lengths (Å)  Bond angles (°) | 0.0140  1.34 | 0.0106  0.93 | 0.0072  0.86 | 0.0136  1.07 | 0.0098  0.97 |
| Validation  MolProbity score  Clashscore  Poor rotamers (%) | 2.14  6.58  1.92 | 2.28  14.86  0.64 | 2.01  10.07  0.46 | 2.16  13.73  0.68 | 1.92  7.94  0.67 |
| Ramachandran plot  Favored (%)  Allowed (%)  Disallowed (%) | 89.59  10.01  0.40 | 87.88  11.64  0.48 | 92.00  7.83  0.17 | 91.32  8.49  0.19 | 91.86  7.93  0.21 |
