# Supplementary material for: Structure and function of yeast Lso2 and human CCDC124 bound to hibernating ribosomes
Source: PLoS Biol. 2020 Jul 20;18(7):e3000780. doi: 10.1371/journal.pbio.3000780 (PMC7392345; doi:10.1371/journal.pbio.3000780)
Supplement: Attachment — Submitted filename: PLOSRebuttal_final.docx [file pbio.3000780.s016.docx]

**RESPONSE TO REVIEWERS:**

Reviewer #1:

        The study by Wells and Buschauer et al. from the Beckmann laboratory reports on the cryoEM structure-based discovery of conserved proteins that stabilize inactive or "hibernating" ribosomes from yeast to humans. These structures are important because they reveal, in molecular terms, how yeast and human cells adapt to fluctuating environmental nutrient availability by reversibly decreasing protein synthesis while simultaneously protecting and maintaining inactive ribosomes for future use. These new structures are insightful and help explain genetic and biochemical data from prior reports about Lso2/CCDC124. The structures also reconcile previous work on an alternative hibernation factor, Stm1/SERBP1, by defining the different ribosome conformations that Lso2/CCDC124 versus SERBP1 stabilize. Biochemical data, including mass spectrometry, confirm the co-purification of CCDC124 and EBP1 with hibernating ribosomes. The structural finding that EBP1, moreover, occludes the surface of the 60S near the exit tunnel is novel and will serve as a useful reference for future functional studies. The structural work is outstanding and thoroughly described. My only question concerns the ribosome-splitting assay and its interpretation.

Major comments

1. The authors predicted that Pelota/Hbs1/ABCE1 will dissociate hibernating ribosomes that are stabilized by Lso2/CCDC124. Specifically, they claim that Pelota/Hbs1/ABCE1 splitting will recycle Lso2/CCDC124-protected ribosomes—but not SERBP1-protected ribosomes—because of the different conformational states seen in their structures. To test whether Lso2/CCDC124 stabilize non-rotated 80S ribosome in a splitting-competent, and therefore recycling-competent state, the authors compare puromycin splitting (control) with Pelota/Hbs1/ABCE1 splitting of Lso2- versus Stm1/SERBP1-engaged 80S ribosomes. By employing a series of controls (omitting nucleotides, omitting individual splitting factors), the authors observe that Lso2-reconstituted ribosomes are split efficiently in vitro, while Stm1-reconstituted 80S ribosomes are not. There is a fly in the ointment though, because "splitting was observed in reactions when all components (eIF6, ABCE1, Hbs1, Dom34, 1mM ATP, 1mM GTP) were present, or in the absence of Hbs1." Shouldn't the absence of Hbs1 have prevented splitting in this negative control?

Response:

We thank the referee for raising the question whether splitting of 80S should be prevented by the absence of Hbs1. Yet, previous studies using very similar in vitro splitting systems showed that splitting is possible in absence of Hbs1. In the study of Shoemaker et al., PNAS 2011, for example, splitting of elongation complexes was already efficient with Dom34 and ABCE1, but addition of Hbs1 and GTP increased the rates. Similar observations were made in the study of van den Elzen et al. NSMB 2014 in which vacant yeast 80S were used. Hbs1 is responsible for the enzymatic delivery of Dom34/Pelota into the A-site of the 80S ribosome very similar to amino-acyl-tRNAs delivery by eEF1A. Whereas this delivery step is functionally relevant in vivo, it is apparently not essential under in vitro conditions when a high molar excess of splitting factors over the ribosome is used. It can be considered to be analogous to the well-known non-enzymatic delivery of tRNA to ribosomes.

We addressed this point in the main text (page 16, line 431ff):

‘Splitting of Lso2-80S was dependent on the presence of nucleotides (1 mM ATP, 1mM GTP), as well as Dom34 and ABCE1*,* consistent with previous studies *(Shoemaker et al, PNAS 2011, van den Elzen et al., NSMB 2014).* Also consistent with these studies is the observation that Hbs1, although required *in vivo,* was not required for ribosome splitting *in vitro.* This indicates that indeed the enzymatic activity of ABCE1 in concert with Dom34 binding to the A site was required to split Lso2-bound 80S ribosomes (S10 Fig).’

2. The comparison with Stm1-containing yeast 80S ribosomes obtained after glucose starvation is valuable, but the comparison does introduce confounding variables because these Stm1-80S particles were obtained from glucose starved yeast rather than reconstituted from subunits and pure Lso1. The authors may want to emphasize that these ribosomes are an imperfect control because they differ from the Lso2-reconstituted ribosomes in many ways, including unknown co-purifying factors or unknown modifications, etc. So although the Stm1-80S complexes were found to be "essentially resistant to splitting by the Dom34 system" this may be due to other differences in the Stm1 particle, not just its rotation status.

Response:

We agree with the reviewer, that the Stm1-80S obtained after glucose starvation may not be readily comparable to the *in vitro* reconstituted Lso2-80S, and, as suggested, emphasize this in the main text (page 17, line 456ff)

*‘Notably, Stm1-80S complexes purified after glucose starvation may not be directly comparable to in vitro reconstituted Lso2-80S complexes. Therefore, we cannot entirely exclude other differences than the rotation status of Stm1-80S contributing to the splitting resistance. Thus,* to check if the splitting-resistant fraction in the Stm1-80S population contained Stm1 bridging the 40S and 60S *in a rotated conformation*, we performed cryo-EM…’

Reviewer #2:

This manuscript reports structural and biochemical evidence that Lso2 stabilizes an inactive ribosome confirmation poised for reactivation by ribosome rescue and recycling factors Dom34 and Rli1. Structures of inactive yeast ribosomes with Lso2 bound are presented, with reconstituted addition of purified Lso2 and native purification from growth-limited yeast appearing very similar. These ribosomes resemble a post-translocation, non-rotated conformation, with Lso2 occupying the peptidyl-tRNA site and interfering with tRNA or factor binding. A fraction of ribosomes from quiescent cultured human cells show a similar non-rotated quiescent state bound to Ccdc124. These human cells also contained inactive ribosomes bound by Serbp1 and eEF2. Further, both of these populations of inactive ribosomes were bound by Ebp1, which interacts near the exit tunnel of the ribosome at a similar site to many co-translational chaperones. The yeast orthologue of Ebp1, Arx1, has a well-supported role in large subunit biogenesis and nuclear export, and these data suggest functional differences between the yeast and human proteins.

        The non-rotated conformation of Lso2 / Ccdc124-bound ribosomes and the partially accessible A site led to the hypothesis that these ribosomes might be poised for reactivation by Dom34*Hbs1. This complex, working in conjunction with the recycling factor Rli1, dissociates subunits of stalled ribosomes. Here, it is shown that Dom34 and Rli1 together split Lso2-bound non-rotated ribosomes, but not Stm1-bound rotated ribosomes. From these results, it is inferred that Lso2-bound ribosomes can be rapidly reactivated by splitting, offering an explanation for the importance of Dom34 in resuming growth from quiescence.

        The high-quality structural and biochemical data here advance our understanding of ribosome storage and reactivation in eukaryotes. Further, this work points towards a possible functional significance for the Lso2-bound ribosome pool that is consistent with genetic evidence. I would support the publication of this manuscript in PLoS Biology, provided the following points are addressed:

1. Other recent studies and pre-prints analyze the structure of Ebs1 on human ribosomes and reach slightly differing conclusions. Wild et al., (Nat Commun 2020) present a structure that seems to share key features with the present study and suggest that Ebs1 blocks the exit tunnel. Kraushar et al. (bioRxiv 2020.02.08.939488) present structural evidence that Ebs1 associates with translating ribosomes. They also argue that Ebs1 is very high abundance at least in certain cell types, and might associate with a substantial fraction of all ribosomes, active or inactive. Ebs1 structures should be discussed in light of these other results, as should the compatibility of Ebs1 with active translation.

Response:

We agree with the reviewer that these very recently published studies should be integrated in the discussion. Here, we also integrated a few points raised by reviewer 4 concerning the role of ES27 which is coordinated by EBP1 on the exit site. We thus modified the paragraph discussing the EBP1 structure. It reads now as follows (page 19 line 500ff):

‘Independent of the ribosomal state and binding of hibernation factors SERBP1-1 or CCDC124, we observed EBP1 bound to the peptide tunnel exit of hibernating human ribosomes. *Our EBP1-80S structure largely resembles recently published structures of EBP1 bound to puromycin-treated 80S ribosomes (Wild et al., Nat. Comms 2020) and of EBP1-bound ribosomes from the mouse neocortex (Kraushar et al., bioRXiv, 2020).* The binding mode we observed is similar to that of other exit site binding proteins which interact with the nascent chain. As a result, this binding mode excludes interaction of the majority of co-translationally acting factors to the ribosome, consistent with a role as hibernation factor that prevents unproductive sequestration of these factors to idle ribosomes. *Interestingly, the study by Kraushar et al., showed that EBP1 binds not only idle 80S but also to translating polysomes (Kraushar et al., bioRXiv, 2020). Here, EBP1 was found highly enriched in the cytosol of early born neuronal stem cells of mouse neocortex where it has been suggested to play a role in modulating protein homeostasis, possibly by competing with other exit site factors. In this context, it is intriguing that EBP1 stabilizes the otherwise flexible rRNA expansion segment ES27 in an exit site- facing conformation. While no data are available for the human system, ES27 is essential in yeast and was shown to directly coordinate and position nascent chain interacting factors such as NatA (Knorr, NSMB) on the exit site*. Another potential role of EBP1 may be in preventing ubiquitination of the ribosomal protein uL29 of the inactive ribosome, thereby contributing to the coordination of its propensity for ribophagy (51). Yet, the exact circumstances and mechanism of how EBP1 is recruited to dormant *and also translating* ribosome*s* and what triggers its dissociation before or during the translation cycle remain to be elucidated.’

2. The data presented here do pose some questions relative to the genetic results regarding Dom34. The contrast between in vitro and in vivo requirements for Hbs1 are discussed in den Elzen et al. (ref 15 of this manuscript) and probably merit mention here. More puzzling, though, why does the loss of Stm1 reduce dependency on Dom34 activity, and overexpression of Stm1 increase dependency on Dom34 activity?

Response:

As already mentioned in our response to reviewer 1, who raised a similar point, we now explained the difference in Hbs1 requirement for the splitting reaction *in vitro* (where Hbs1 is not required in case of a molar excess of Dom34 and ABCE1 over ribosomes) and *in vivo* (where Hbs1 is required for efficient recovery after glucose starvation, most likely by promoting Dom34-dependent splitting). We changed this paragraph in the results part (page 16, line 431ff):

:

‘Splitting of Lso2-80S was dependent on the presence of nucleotides (1 mM ATP, 1mM GTP), as well as Dom34 and ABCE1*,* consistent with previous studies *(Shoemaker et al, PNAS 2011, van den Elzen et al., NSMB 2014).* Also consistent with these studies is the observation that Hbs1, although required *in vivo,* was not required for ribosome splitting *in vitro.* This indicates that indeed the enzymatic activity of ABCE1 in concert with Dom34 binding to the A site was required to split Lso2-bound 80S ribosomes (S10 Fig).’

Additionally, we slightly rephrased the discussion when reporting about the requirement of Dom34 as well as Hbs1for recovery after glucose starvation (van den Elzen, NSMB 2014).

Regarding the question why the loss of Stm1 reduces dependency, and overexpression of Stm1 increases dependency on Dom34 activity, we already tried to refer to the arguments from the discussion in the van den Elzen paper, yet we apologize to be unclear here. Basically, the authors argue that even in non-stressed cells, Stm1 is bound to ribosomes in a form antagonizing the Dom34-Hbs1 splitting system. When deleting both Stm1 and Dom34, they observe a lower fraction of 80S in polyribosome gradients than when only deleting Dom34 (compare Figures 1b and 3b in their paper). This indicates that due to the absence of Stm1 a larger pool of ribosomes, most likely completely idle ones, which don’t require Dom34/Hbs1 for splitting, is available. Along those lines, if Stm1 is overexpressed, this pool of “spontaneously” dissociating 80S is smaller, and if then Dom34 is also deleted (to split the remaining idle 80S) cells develop a growth defect. We tried to summarize this line of arguments a bit clearer in the revised manuscript. The paragraph in the discussion now reads as follows (page 20, line 555ff):

‘Along the same lines, deletion of the stabilizing Stm1 suppresses the requirement of Dom34 and Hbs1 in recovery of translation(15). *Here, a likely explanation is, that in the absence of Stm1 there is a larger pool of idle ribosomes that are unstable and can be easily recycled. Thus, recovery of translation depends to a lesser extent on the recycling of ribosomes that strictly require the Dom34 system for splitting.* In support of *this antagonism between Stm1 and Dom34* overexpression of Stm1 in a *dom34Δ* strain leads to a growth defect, likely due to an accumulation of Stm1-80S and the inability to split the remaining pool of 80S by the Dom34 system.’

3. As a minor point, p. 10 l. 183, "Two distinct, inactive ribosomal species are present IN eukaryotes"

Response: We corrected this.

 Reviewer #3:

        The mechanisms by which ribosomes are idled in the cell during periods of non-proliferation have been extensively examined in bacteria for multiple decades. Recently, high-resolution structural information has been revealed that shed light on the multiple distinct mechanisms for idling that exist by which the cessation of ribosome activity can be achieved. This includes a long-term storage mechanism that generates 100S "disosomes". Such mechanism play critical roles in bacterial physiology and fitness.

        The present investigations are very important as the add to the emerging understanding of ribosome-arrest and hibernation in eukaryotic (yeast and human) cells about which very little is known. The structural and biochemical investigations presented suggest that the complex of Lso1, a protein recently discovered in yeast, with the ribosome has the capacity to bind in a manner that may equips them to be recycled by the "splitting factor" Dom34. The authors attribute this capacity of Lso1s and the human homologue CCDC124's to stabilization of the 80S ribosome in an unrotated conformation, which is competent for Dom34 binding and activity.

        The authors elegantly substantiate the importance of this finding though parallel structural and biochemical investigations of  arrested 80S ribosome complex that are bound to either protein factors, smt1 (yeast) or SERBP1 (human), which stabilize ribosomes in a rotated conformation, which is recognized and further stabilized by eEF2 and tRNA binding within the E site. In carefully executed, appropriately integrated structural and biochemical assays, the authors provide what appears to be concrete evidence that Dom34 is simply unable to engage the rotated ribosome conformation that is stabilized by stm1/SERBP1 even though there should be adequate space to do so in the absence of eEF2. These findings provide a compelling and complete story that reveals the existence of distinct types/classes of arrested 80S complexes and opens the door for numerous downstream investigations aimed at exploring the physiological roles of such complexes in distinct tissues and cellular environments and the regulatory mechanisms and timing that control the formation and disassembly of such arrested complexes.

        The manuscript is very clear and well written, the figures efficiently convey the findings and the data strongly support the major conclusions drawn. There are multiple high-resolution structures presented: some have not been observed previously; others are reported and higher-resolution that analogous complexes reported previously. For this reason I recommend publication.

        The questions that were raised for me during review, which the authors may wish to address relates to the **cellular concentrations of Lso1**, **stm1 and SERBP1** and how this may tie into the distinct regulatory mechanisms that they propose. Are these factors repressed under normal, proliferative conditions and somehow activated during stress?

Or are they always present in the cell at relatively high concentrations? Based on my understanding of the authors discussion, Initiation is where they suggest Lso1/CCD124-mediated regulation manifests. However, the Dom34 studies suggest that Lso1/CCD124 functions may also manifest during translation elongation, correct? Impacts on elongation would require Lso1/CCD124 binding to actively translating ribosomes. Is this the correct inference?

Whether it be initiation or elongation (or both), the observed pose of Lso1/CCD124 on the ribosome also raises the question of how these proteins bind in the first place. The structures identifies that Lso1/CCD124 are positioned on the 80S ribosome in a manner that precludes tRNA binding at both the P and A sites. Importantly, one or both of these tRNA binding sites would largely be occupied during scanning initiation steps, 80S initiation complex formation and translation elongation. Does Lso1/CCD124 stimulate tRNA release (at least P site tRNA release)?

It would seem prudent to speculate on this aspect of the proposed model in the discussion section as it would seem relevant to specifying which population of ribosomes in the cell are indeed targeted.

We agree that the reviewer is raising valid questions with respect to the regulatory mechanisms underlying the Lso2/CCDC124 ribosome interaction, because for some bacterial hibernation factors it is well documented that their expression is repressed during proliferative conditions and induced upon stress. However, what is known about the eukaryotic systems hints towards a different mode of regulation:

(i) Regarding cellular concentrations in yeast, according to the Saccharomyces Genome Database (SGD), Stm1 is close to ten times more abundant than Lso2 (approx. 70000 copies per cell for Stm1 vs 8000 per cell for Lso2) and thus it is in the range of ribosomal proteins.
(ii) Regarding regulated expression not much is known. Notably, and in contrast to the bacterial hibernation factors, the available data indicate that Stm1 as well as Lso2 are always present and are not suppressed during proliferation and up-regulated by stress (Wang et al., 2018).
(iii) Regarding whether the Lso2/Dom34 regulation is manifested during initiation or elongation, the reviewer is correct that it is initiation in the first place, because efficient splitting of idle 80S would as immediate result provide 40S subunits for initiation. We do not believe, and there is no evidence, that Lso2/CCDC124 or Stm1/SERBP1 can directly interfere with elongation (for example by competing with mRNA or tRNA in elongating ribosomes), although Stm1 has indeed been found associated with active polysomes.
(iv) Regarding the question how the hibernating complexes are formed, unfortunately nothing is known so far.
(v) And there is also no data on the question whether Lso2/CCDC124 can actively stimulate tRNA release.

For eukaryotes it is well established that different kinds of stress result first in a global shutdown of translation, which in turn results in increasing numbers of idle subunits and 80S. These, we speculate, are the main target of Lso2/CCDC124 or Stm1/SERBP1. Therefore, since so little is known about how these complexes are formed, we would like to refrain from further speculations in the Discussion.

        Another curious observation made by the authors, which may be related somehow, relates to the observed binding of EBP1 to the exit tunnel of their arrested complexes. The observed occupancy levels in their samples, and the positioning of this protein at the exit tunnel so as to block the binding of chaperones and other active protein synthesis interacting proteins, would suggest that Lso1/CCD124 bind ribosomes that have somehow already terminated translation. In this model, both proteins may bind so as to ensure that they are efficiently halted to prevent further activity or that they are shunted to recycling to ensure downstream initiation events rather than being employed elsewhere or degraded. Either way (or other ways) some mention should be made about what triggers EBP1 binding to the arrested ribosomes. It may also be worth mentioning in this context the potential impacts of sequestering ES27 near the exit tunnel as Figure 4A would appear to suggest. Is EBP1 also present at high concentrations in the cytoplasm? Does EBP1 simply have the tendency to bind ribosomes that don't have other factors bound (ie. perhaps lacking nascent peptides). The authors state EBP1 a ribosome biogenesis factor, which would suggest nucleolar localization. Is there any information out there on its expression levels and/or cytoplasmic concentration?

        To be clear, the inclusion of such details and speculations are just suggestions. If the authors wish to not speculate further, this should by no means prevent the authors from moving forward with reporting this exciting and new set of findings.

Indeed, during the course of submitting this paper, a new study appeared in bioRxiv partly addressing the questions raised by the reviewer. The study by Kraushar et. al shows that EBP1 is cell-type specific and is highly expressed in early-born neural stem cells in the mouse neocortex. In this paper, the authors show that EBP1 exists in two splice variants and that the full-length EBP1 is mainly localized in the cytosol. Moreover, they show by cryo-EM that EBP1 interacts with the translation machinery in a very similar way as presented in our work. It shows in addition, that EBP1 binds not only to idle but also to translating ribosomes and thus may be able to modulate protein homeostasis by competing with nascent chain interacting factors. As also proposed by reviewer 2, we integrated the most important aspects of Kraushar et al. in our discussion.

Regarding the involvement of ES27 we included the point in the discussion that ES27 – at least in yeast – was shown to be involved in positioning and coordinating nascent chain interacting exit site factors. This indeed may play a role in the context of regulation of gene expression as presented in Kraushar et al.

As already pointed out in the response to reviewer 2, our modified discussion now reads as follows (page 19 line 500ff):

‘Independent of the ribosomal state and binding of hibernation factors SERBP1-1 or CCDC124, we observed EBP1 bound to the peptide tunnel exit of hibernating human ribosomes. *Our EBP1-80S structure largely resembles recently published structures of EBP1 bound to puromycin-treated 80S ribosomes (Wild et al., Nat. Comms 2020) and of EBP1-bound ribosomes from the mouse neocortex (Kraushar et al., bioRXiv, 2020).* The binding mode we observed is similar to that of other exit site binding proteins which interact with the nascent chain. As a result, this binding mode excludes interaction of the majority of co-translationally acting factors to the ribosome, consistent with a role as hibernation factor that prevents unproductive sequestration of these factors to idle ribosomes. *Interestingly, the study by Kraushar et al., showed that EBP1 binds not only idle 80S but also to translating polysomes (Kraushar et al., bioRXiv, 2020). Here, EBP1 was found highly enriched in the cytosol of early born neuronal stem cells of mouse neocortex where it has been suggested to play a role in modulating protein homeostasis, possibly by competing with other exit site factors. In this context, it is intriguing that EBP1 stabilizes the otherwise flexible rRNA expansion segment ES27 in an exit site- facing conformation. While no data are available for the human system, ES27 is essential in yeast and was shown to directly coordinate and position nascent chain interacting factors such as NatA (Knorr, NSMB) on the exit site*. Another potential role of EBP1 may be in preventing ubiquitination of the ribosomal protein uL29 of the inactive ribosome, thereby contributing to the coordination of its propensity for ribophagy (Brandmann and Hedge, 2016). Yet, the exact circumstances and mechanism of how EBP1 is recruited to dormant *and also translating* ribosome*s* and what triggers its dissociation before or during the translation cycle remain to be elucidated.’

Reviewer #4:

This work by Wells focuses on the structural studies of the hibernating ribosomes from yeast and human. The authors determined several cryo-EM structures at a very good resolution of the yeast 80S ribosome with a new hibernation factor Lso2 and human 80S ribosome in complex with protein CCDC124, which is homologous to Lso2. Also, the authors determined a new structure of the human 80S ribosome in complex with alternative hibernation factor SERBP1. This work represents a long-awaited continuation of the previous work by the same and other groups and reveals many interesting structural aspects of ribosome hibernation in eukaryotes. Studying the mechanisms of ribosome hibernation and the protein factors mediating this process in eukaryotes is crucial not only from a fundamental point of view but also has far-reaching medical implications because many of these hibernation factors are related to cancer development.

        One of the most interesting findings of this work is that eukaryotes exploit two distinct mechanisms for ribosome hibernation: one employs proteins Lso2/CCDC124 and preserves 80S ribosomes in the unrotated state, while the other utilizes proteins Stm1/SERBP1 and traps the ribosomes in the ratcheted state. This is quite distinct to the mechanisms of ribosome hibernation operating in bacteria. Also, this work highlights how distinct and diverse are the ribosome hibernating factors from pro- and eukaryotes. In addition to quite intense structural studies, the authors confirmed some of their ideas and hypotheses with biochemical in vitro experiments using purified Dom34 and other ribosome splitting factors. I find the results of the ribosome splitting experiments and subsequent structural analysis of some of the complexes as "a cherry on the cake" that definitely reinforces the whole story.

        Overall this work is an excellent and exemplary research study that was accomplished in the best traditions of the modern cryo-EM structural biology! In my opinion, this work represents a significant conceptual advance in the field and also answers several long-standing questions. I think that the main findings of this study merit publication in PLOS Biology and are ideally suited for this journal in scope. Moreover, the manuscript is very well-written and organized. There are simple and bright illustrations that are self-explanatory. It appears to me that the text and figures have already been thoroughly revised, and it seems to be close to its energetic minimum. In conclusion, I am enthusiastically in favor of publishing this work. Also, I have a couple of minor critical points/suggestions, which the authors might wish to address:

        Comments, suggestions, and questions to the authors:

1. Lines 325-328: I would like to suggest to the authors to revise this sentence to read something like "However, while bacterial RMF, SHPF, RaiA (protein Y) or the LHPF N-terminal domain all target the mRNA path and the decoding center of the small subunit, eukaryotic Lso2 and CCDC124 bind to both, the 40S and 60S subunits simultaneously and exclusively stabilize the non-rotated conformation of the 80S ribosome."

        SHPF - short HPF, LHPF - long HPF. I am not sure what the authors meant by IHPF? Perhaps it was just a typo. Also, RaiA is better known as ribosomal protein Y, so maybe somehow include this name as well?

Response: IHPF was a typo and we corrected it, as pointed out correctly by the reviewer, to LHPF. We also included the name “protein Y” (pY) for Rai1.

2. Figure 5, panels C and D: Why the positions of the peaks corresponding to the unsplit 80S ribosomes in panel C do not match the same peaks in panel D (except for the plots with Stm1)? In other words, the 80S peaks at the top and bottom plots should have the same positions relative to the gradient start.

Response: The fact that the 80S peaks in these figure panels are not matching is due to the alignment, since sucrose density gradient centrifugations were performed on different days and differently appearing void peaks were cut off. We agree with the reviewer, that aligned profiles are easier to read and we adjusted the profiles and aligned them to the 80S peak (see revised Fig 5).
